# Did outside directors’ firm-specific accumulated knowledge benefit the firm’s stock performance during COVID-19?

**DOI:** 10.1177/03128962231166831

**Published:** 2023-04-29

**Authors:** Dharmendra Naidu, Kumari Ranjeeni

**Affiliations:** Department of Accounting, Monash Business School, Monash University, Clayton, VIC, Australia; Pra^2^Njeeni, Berwick, VIC, Australia

**Keywords:** Accumulated knowledge, COVID-19, director tenure, excess stock returns, global crisis, social capital

## Abstract

We examine whether outside directors’ firm-specific accumulated knowledge in the forms of human and internal social capital benefitted the firm during COVID-19. Using a sample of 754 US firms during the COVID-19 collapse period, we find an inverted U-shaped relation between outside directors’ average board tenure and cumulative excess stock returns. Our result suggests that firms experienced optimal cumulative excess stock returns during COVID-19 when outside directors’ average board tenure is 10 years. We also find that the curvilinear relation is profound for outside directors with more internal social capital, suggesting that outside directors’ internal social capital plays a prominent role in enhancing board effectiveness during a crisis. Furthermore, we use several robustness checks to confirm the results.

**JEL Classification:**

D83, G30, G34, M41

## 1. Introduction

Recent reports suggest that boards can play an influential role to guide businesses through a crisis (Cohn, 2020; Sutherland, 2020; Von Post and Pozen, 2020).^1^ However, the relevance of outside directors’ accumulated knowledge about a firm to decision-making during the COVID-19 crisis is unknown. Recent research examines the effects of government intervention to circumvent the detrimental impact of COVID-19 (e.g. Naidu and Ranjeeni, 2021; Narayan et al., 2020; Zaremba et al., 2020). Other studies examine the effects of firm characteristics on stock returns during the crisis (Fahlenbrach et al., 2021; Ramelli and Wagner, 2020).

There is a lack of research on how corporate governance strategies may have benefitted firms during COVID-19 (e.g. Ding et al., 2021). Since COVID-19 is a health crisis, scholars (e.g. Zattoni and Pugliese, 2021) argue that corporate governance-related research findings from previous financial crises may not be directly generalizable to the COVID-19 crisis and call for future research to use firm-specific secondary data to examine the effect of corporate governance on firms during COVID-19 crisis (Jebran and Chen, 2023; Koutoupis et al., 2021). We respond to this call and are motivated to examine whether outside directors’ firm-specific accumulated knowledge benefits the firm’s stock performance during COVID-19.

Drawing from human and social capital theory and consistent with prior studies (Brown et al., 2017; Huang and Hilary, 2018), we argue that outside directors with more firm-specific knowledge are more resourceful for a firm in responding to COVID-19 challenges because they are potentially more aware of the firm’s underlying core competencies. However, the COVID-19 pandemic has introduced new challenges for board of directors that is also different from the challenges imposed by prior financial crises.

The COVID-19 pandemic is a unique global health crisis (Jebran and Chen, 2023). It imposed new challenges for corporate boards to function effectively and for them to effectively guide their businesses through the crisis. We argue that the unique challenges imposed by COVID-19 such as pandemic, mobility restrictions and lockdowns (Zattoni and Pugliese, 2021) may have affected the behaviour of board of directors differently in comparison to normal times or prior financial crises by creating constrains on the ability of directors to utilize their accumulated knowledge in the following ways. For instance, due to lockdowns, board members had to meet virtually (online) as opposed to face to face. Such a sudden switch to virtual meetings may have adversely affected board interactions due to process-losses and lower cognitive conflicts and dissent (Zhu, 2013). Some directors may have been camera shy. This would have adversely affected their behaviour and, hence, their concentration or decision-making abilities during virtual meetings.

In addition, a rapid rise in the rate of COVID-19 deaths and infections may have imposed challenges not only to firm’s risk management strategies but also at an individual director level. Directors’ thought processes may have been affected as the uncertainty to human lives created panic in the society and fear for life (either their own life or the lives of someone near and dear to them). These collectively may have impacted directors’ decision-making. Therefore, the effect of outside directors’ accumulated knowledge about a firm on decision-making during the COVID-19 crisis is empirically unclear.

To the best of our knowledge, our research is the first to examine the effect of outside directors’ firm-specific human and internal social capital on stock returns during COVID-19. We draw from human and social capital theory and specifically focus on firm-specific human capital and internal social capital of outside directors (Burt, 1992; Fischer and Pollock, 2004; Grant, 1996; Kor and Sundaramurthy, 2009). Outside directors accumulate knowledge about a firm over their tenure. We argue that firm-specific knowledge would have been very resourceful for outside directors in quickly and competently responding to COVID-19 challenges because they would potentially be more aware about the firm’s underlying core competencies, have more knowledge and confidence in making rapid decisions during COVID-19 and provide timely advice to the management team.

Based on the theory of social capital, we argue that prior social interactions between outside directors and inside directors over their tenure (we refer to this as outside directors’ internal social capital) may have been useful during the COVID-19 crisis. Executive directors have better knowledge of their business and industry context (Zattoni and Pugliese, 2021). Outside directors’ internal social capital may have enabled them to access soft information from inside directors during the COVID-19 crisis as lockdowns and mobility restrictions affected information flow to outside directors from other sources. This may have contributed to relevant and rapid decisions being made during the COVID-19 crisis. Outside directors’ internal social capital may have also contributed to better functioning of board-management as a team during COVID-19 via better understanding and trust of each other developed over time.

We find an inverted U-shaped relation between outside directors’ average board tenure and cumulative excess stock returns during the COVID-19 collapse period. The result suggests that firms with outside directors with a higher accumulated firm-specific knowledge experienced a lower decline in cumulative excess stock returns during the COVID-19 collapse period, where outside directors’ average board tenure is less than or equal to 10 years. However, we do not find a significant relation between outside directors’ average board tenure and cumulative excess stock returns during the COVID-19 collapse period for firms with the tenure more than 10 years. Our results suggest that outside directors’ firm-specific knowledge accumulated over their board tenure was beneficial for the firm in responding to the challenges posed by COVID-19. In economic terms, our results suggest that an increase in outside directors’ average board tenure from four to eight years resulted in a 17.15% increase in cumulative excess stock returns during the COVID-19 collapse period.^2^

Next, we find that the relation between outside directors’ average board tenure and cumulative excess stock returns during the COVID-19 collapse period is significant only for firms with a higher proportion of inside directors and inside directors with a longer average board tenure. This result suggests that outside directors’ internal social capital accumulated through their interactions with more inside directors over a longer tenure was beneficial for them in utilizing their firm-specific human capital to contribute to board effectiveness in responding to the crisis. In economic terms, for outside directors with a higher internal social capital, the findings suggest that an increase in their average board tenure from 4 to 8 years resulted in a 23% to 29% increase in cumulative excess stock returns during the COVID-19 collapse period.

In our empirical model, we control for other board characteristics and firm characteristics. For robustness checks, we use the event study methodology where firm stock performance is measured as the cumulative abnormal returns based on the market model. The results are consistent with our main findings. Next, we test the sensitivity of our results by excluding firms from industries that are most represented in the sample and affected by COVID-19. We exclude healthcare, business services, retail, machinery, petroleum and natural gas, and electronic equipment industries and continue to find supportive results. We further confirm that our results are robust upon using a propensity score-matched sample. In our final additional analyses, we measure firm stock performance for alternative time periods during the COVID-19 pandemic. The results collectively suggest that outside directors’ firm-specific accumulated knowledge has been beneficial for the firm’s stock performance during the COVID-19 pandemic.

Our study makes the following contributions. First, we contribute to the scarce literature on the role of corporate governance in enhancing firm performance during a crisis (e.g. Ding et al., 2021; Francis et al., 2012). Using samples from a non-crisis period, recent studies suggest that investors value directors’ on-the-job learning up to a threshold (Brown et al., 2017; Huang and Hilary, 2018). However, crisis imposes critical challenges for firms (Lin et al., 2006), and hence, similar corporate governance practices may not be effective during both normal and crisis periods (Jebran and Chen, 2023). Prior research suggests that theories of corporate governance may not apply globally in all situations (Judge, 2012). Coronavirus affected boardroom dynamics by abruptly shifting face-to-face meetings to virtual ones (Zattoni and Pugliese, 2021). Our research is the first to show how prior social interactions between outside directors and inside directors over their tenure positively contributed to firm performance during the COVID-19 crisis.^3^ We contribute by advocating on the relevance of outside directors’ accumulated knowledge about a firm to decision-making during the COVID-19 crisis.

Second, we contribute to the literature on the implications of outside directors’ accumulated knowledge of a firm as proxied by their board tenure. Vafeas (2003) makes the first attempt towards understanding the complex issue of the full effect of board tenure. Vafeas (2003) highlights that identifying the appropriate length of board tenure is interesting and relevant to public policy about corporate boards. Recently, Huang and Hilary (2018) show an inverted U-shaped relation between board tenure and firm performance using a sample of US firms for the period 1998–2010. Brown et al. (2017) suggest that the shareholder-assessed value of directors varies with the tenure of individual director and they also show an inverted U-shaped relation using a sample of US firms for the years 2000–2012. We extend this strand of literature by examining the effect of outside directors’ internal social capital on their utilization of firm-specific human capital during the COVID-19 collapse period.

Third, our study has practical implications for the debate on whether regulators should consider a limit on outside directors’ board tenure due to the trade-off between accumulated knowledge and managerial entrenchment. Our results add to the findings of prior studies (Brown et al., 2017; Huang and Hilary, 2018) that a limit on board tenure may benefit firms even during a crisis. Brown et al. (2017) document an optimal board tenure between 7 and 18 years. Huang and Hilary (2018) suggest an optimal tenure between 8 and 11 years. Our results suggest that outside directors’ board tenure around 10 to 13 years was optimal during the COVID-19 collapse period. Our results are also relevant to regulators and stakeholders of firms in the Asia-Pacific region including Australia because recent studies using Australian firms show that their average board tenure is around 6 years (He et al., 2020; Sun et al., 2022).

Finally, our findings also have implications for the debate on the trade-off between board independence and management friendliness (e.g. Adams and Ferreira, 2007; Schmidt, 2015). Our results suggest that outside directors’ internal social capital accumulated through their interactions with inside directors is valuable during a crisis. The results imply that a board that utilizes outside directors’ accumulated knowledge and relation with inside directors is optimal for board effectiveness and stock performance during a crisis. Our results support the findings of Schmidt (2015) who suggests that management-friendly boards improve board quality through information exchange when board advice is important.

## 2. Theoretical framework and hypotheses

### 2.1. Outside directors’ accumulated knowledge and firm performance

Anecdotal evidence suggests that board of directors’ experience may be useful in times of crisis. For instance, Sutherland (2020) suggests that the collective wisdom and experience of directors is important to shape a firm’s response to a crisis. Cohn (2020) in a Forbes article reports that ‘board needs to be available as a group or individually to provide advice and counsel based on their expertise and past experience’. We, therefore, argue that outside directors’ experience in serving at a particular firm’s corporate board would have benefitted the firm during the COVID-19 crisis.

Our argument is based on the theory of human capital (Brown et al., 2017; Kor and Sundaramurthy, 2009). Each outside director begins serving on a board with limited firm-specific knowledge, like new outside CEOs (Hambrick and Fukutomi, 1991). Over their tenure, outside directors accumulate firm-specific knowledge, such as a firm’s prior commitments, unique resources, core competencies, needs, potential sources of growth and boardroom norms (i.e. human capital) (Brown et al., 2017; Kor and Sundaramurthy, 2009). Such firm-specific knowledge may have been very resourceful for outside directors’ in responding to COVID-19 challenges because they would potentially be more aware about the firm’s underlying core competencies.

The COVID-19 pandemic, lockdowns, and mobility restrictions created new challenges for corporate boards as businesses had to be conducted differently and this involved tactical decision-making. Shift to online business put pressure on customer experience. For instance, in a podcast by McKinsey & Company (2021), Bisson (2021) states that ‘the test for the board is, can you keep your eye on the key strategic decisions or transformations you are pursuing, recognizing that management may be reaching the saturation point in what it can manage?’ As such, outside directors could have utilized their firm-specific knowledge to provide relevant and effective advice to the management team in guiding their business through the COVID-19 crisis.

Supply chain disruptions and panic buying during the pandemic put ‘enormous pressure in terms of staffing, supply chain, and keeping food on the shelf, so having an engaged, experienced, and industry knowledgeable board is critical. This is not a time for learning’. (Aufreiter, 2021). Outside directors who have more firm-specific experience would have more knowledge and confidence in making rapid decisions during COVID-19 and providing timely advice to the management team.

A rapid rise in the rate of COVID-19 deaths and infections has imposed challenges to risk management strategies. After lockdowns, businesses had to adhere to Government rules and make provisions for a COVID-19-safe environment, for instance, by enabling social distancing and making provisions for hand sanitization. To quickly and competently respond to these challenges, it is necessary for outside directors to have a basic understanding about the firm’s core competencies so that they can accordingly strategize.

Moreover, Jebran and Chen (2023) advocate the importance of board independence during the COVID-19 crisis. ‘Independent directors, by virtue of their expertise and experiences, can increase decision-making efficiency’ (Jebran and Chen, 2023: 3). On the other hand, a stream of prior research discusses reduction in board independence as a potential downside of outside directors with extended board tenure. According to prior research (Brown et al., 2017; Wade et al., 1990; Walsh and Seward, 1990), due to extended board tenure, outside directors may become management friendly and have loyalty for each other and the CEO. Outside directors may become too familiarized with each other, inside directors and management that may undermine board independence (Huang and Hilary, 2018) or potentially accentuate groupthink (Brown et al., 2017). It follows that during the COVID-19 crisis, outside directors with extended board tenure may have become accustomed to a particular view of a firm’s opportunities and challenges, use less external information, avoid debating contentious issues and develop a tendency for groupthink. All these would have adversely affected board performance during the COVID-19 crisis. Thus, our first hypothesis (H1) is as follows:

H1: Outside directors’ average board tenure is curvilinearly related to cumulative excess stock returns during the COVID-19 collapse period.

### 2.2. Interplay between outside directors’ accumulated knowledge and internal social capital

Internal social capital can be built within the board over the tenure of outside directors if they have more interactions with more inside directors. Coleman (1988) discusses three forms of social capital. First, obligations, expectations and trustworthiness are built between outside and inside directors if an outside director provides something to an inside director and trusts that the inside director would reciprocate in the future (Coleman, 1988). Such expectations from outside directors and obligations on the part of inside directors depend on trust between them that is built through their relations developed by serving together on a board. Also, for boards with more inside directors, it is more likely that inside directors’ total obligations to reciprocate is more. In times of a crisis, outside directors can materialize on the accumulated expectations where inside directors could be obliged to return benefits in various forms, including soft and inside information.

Second, outside directors can use social relations built with inside directors as an information channel to acquire current firm-specific information (Coleman, 1988), which can be very useful in times of a crisis. An outside director who has served together with more inside directors for more years on a board is likely to have more information channels. Third, through social relations between outside and inside directors, an effective norm can be developed over their tenure (Coleman, 1988), which could be beneficial during a crisis. Such norms can be powerful and facilitate actions that are aligned to shareholder interests and constrain self-interest actions. For example, on behalf of shareholders, corporate boards advise and monitor managers. Outside directors being independent of managers can potentially uphold the board’s function more effectively. Through their social relations with inside directors during their board tenure, outside directors can contribute to the development of prescriptive norms with inside directors to promote shareholder interests and constrain opportunistic behaviour. Once an effective norm is developed, such norms could be beneficial during a crisis.

During the COVID-19 crisis, corporate boards were required to make rapid decisions to effectively deal with numerous uncertainties surrounding business operations (McKinsey & Company, 2021). ‘A lot of the efficiency in decision making has been driven by a strong board-management interface’ (Bisson, 2021). As such, outside directors may have leveraged their social relations built with inside directors and accessed information from them. This would have been particularly useful during the COVID-19 crisis because lockdowns and mobility restrictions would have affected information flow to outside directors from other sources. In such a situation, we argue that outside directors’ accumulated firm-specific knowledge particularly in the form of internal social capital is valuable for strategic decision-making during the COVID-19 crisis.

Executive directors have profound knowledge of the business and industry (Zattoni and Pugliese, 2021). During the COVID-19 crisis, outside directors would require relevant, reliable and timely information from management to make rapid decisions so that they could appropriately advise managers and guide the business through the crisis. Outside directors accumulated internal social capital may become useful for them in obtaining soft information, understanding rationales behind management proposals and any tendencies for opportunistic actions.

However, a concern is that outside directors extended social interactions with more inside directors could lead to collusion, managerial entrenchment, compromise of board independence or accentuating groupthink, which could either individually or collectively harm board effectiveness during a crisis. This is likely to result in lower cumulative excess stock returns of firms with outside directors having extended board tenure.

We argue that there are two ways in which outside directors could have leveraged their social capital during the COVID-19 crisis. First, by serving on a corporate board for a longer period, outside directors would have accumulated knowledge through observations, management internal reports, interactions with employees and interactions with inside directors. During COVID-19, if long-tenured outside directors required any further information, then they could have contacted inside directors directly. A higher proportion of inside directors on the board during the COVID-19 crisis imply more information sources or channels for outside directors to acquire additional firm-specific information. This leads us to the following hypothesis:

H2a: The curvilinear relation between outside directors’ average board tenure and cumulative excess stock returns during the COVID-19 collapse period is profound for corporate boards with a high proportion of inside directors.

Second, we argue that an outside director who has served together with inside directors for more years on a board is likely to have had more social interactions and built a stronger relationship in terms of trust and understanding. Outside directors could have garnered the aforementioned during the COVID-19 crisis, especially because the virtual nature of the interactions created challenges. According to a board director,

‘Many of the sidebar style interactions between individual executives and board members at the coffee bar, those natural touchpoints do not happen, so I have seen more outreach from management to individual board members or board member to board member’ (Aufreiter, 2021).

A sudden shift from face-to-face meetings to virtual meetings could have resulted in ‘potential risks in terms of process-losses and lower cognatic conflicts and dissent’ (Zattoni and Pugliese, 2021: 1408). In such situations, we argue that prior social interactions between outside directors and inside directors would have contributed to better functioning of board-management as a team during COVID-19 via better understanding and trust of each other. This would have enabled rapid decisions to be made during the COVID-19 crisis. This leads us to our next hypothesis:

H2b: The curvilinear relation between outside directors’ average board tenure and cumulative excess stock returns during the COVID-19 collapse period is profound for corporate boards with inside directors having a longer average board tenure.

## 3. Research design and variable measurement

To examine the effect of outside directors’ accumulated knowledge of a firm on the firm’s stock performance during the COVID-19 collapse period, we follow Huang and Hilary (2018) and estimate the following empirical model (1). Consistent with Huang and Hilary (2018), we use a lead-lag specification to alleviate endogeneity concerns. That is, the dependent variable is measured using data during the collapse period in 2020, and the independent variables are measured for the year ending 2019. We report *t*-statistics that are corrected for heteroscedasticity

(1) $$\begin{array}{l} CumExcRet_{t+1}=\alpha +\beta_{1}IndDirTenure_{t}+\beta_{2}IndDirTenure_{t}^{2}+\beta_{n}CONTROLS_{t}\\ \;\;\;\;\;\;\;\;\;\;\;\;\;\;\;\;\;\;\;\;\;\;\;\;\;\;\;\;\;\;\;+\beta_{k}IndustryFE+\varepsilon_{t+1} \end{array}$$

We estimate ordinary least squares (OLS) regressions of model (1) using our full sample to test hypothesis 1. To test hypothesis 2a (2b), we form terciles of our full sample based on the proportion (average board tenure) of inside directors on the board and estimate OLS regressions of model (1) within each tercile. In the earlier section, we argue that outside directors’ internal social capital can be built over their tenure with more interactions with more inside directors on the board. Hence, our proxies for outside directors’ internal social capital are the proportion of inside directors on the board and the average tenure of inside directors.

$CumExcRet_{t+1}$ represents cumulative excess stock returns during the COVID-19 collapse period. We use the log daily excess returns to compute the cumulative excess stock returns. Log daily excess returns represent the natural logarithm of one plus the stock return less 1-month daily Treasury bill rate. We cumulate the log daily excess returns over the COVID-19 collapse period.

Consistent with Fahlenbrach et al. (2021), we define the COVID-19 collapse period from 3 February to 23 March 2020. 3 February represents the first day after the lockdown in Wuhan ended. The Chinese stock market was closed from 24 January until 2 February.^4^ On 3 February, the market at the close of the day recorded a decline of 7.7% in Shanghai Composite Index (Shan and Tang, 2020). Since news rapidly spread globally with the use of Internet technologies and social media, we focus on stock returns from 3 February 2020. In addition, we end the collapse period on 23 March 2020 because it represents the last day before the effect of the US stimulus package was observed in the stock market. The US Senate considered a stimulus package to revive the economy but voted ‘no’ on 22 March (Associated Press, 2020). On 23 March, the Federal Reserve (2020) announced extensive measures to relieve the financial stress. Although the US stock markets did not respond immediately, it responded positively on 24 March when it learned that the stimulus package was likely to be approved (Reuters, 2020).

We use average board tenure of outside directors as at 2019 fiscal year to proxy for their accumulated knowledge about the firm prior to the COVID-19 pandemic (i.e. IndDirTenure ). IndDirTenure is the average number of years since the outside directors have been serving on the firm’s board. Consistent with recent studies (e.g. Brown et al., 2017; Huang and Hilary, 2018), we include the squared term of IndDirTenure in model (1) to account for the conflicting effects of accumulated knowledge and compromised monitoring. CONTROLS include control variables that proxy for board characteristics and firm characteristics. We also include industry fixed effects (*Industry FE*) because COVID-19 did not affect all industries in a similar fashion. We discuss the definition of all control variables in Appendix 1.

## 4. Sample selection and descriptive statistics

We obtain data for the test variable and board characteristics from Institutional Shareholder Services (ISS). We obtain data to compute busy board from BoardEx. Further, we obtain stock prices and returns data from Compustat Daily Security file and annual report data from Compustat Fundamentals file. Institutional shareholdings data are obtained from Thomson Reuters 13f data files.

Our sample selection procedure, which is reported in Panel A of Table 1, starts with 1586 firms with non-missing data on board characteristics. We delete 442 firms in the Financial (6000 ⩽ SIC ⩽ 6999) and Utilities (4900 ⩽ SIC ⩽ 4999) sectors. Next, we delete 390 firms with missing data on cumulative excess stock returns and control variables. The sample selection procedure results in a final sample of 754 firms. Consistent with accounting and finance studies, we winsorize all continuous variables at their first and 99th percentiles to minimize the effect of any outliers in our analyses.

**Table 1.** Sample selection and distribution.

Panel A: Sample selection procedure		
		Firms
Initial observations with data merged from COMPUSTAT, ISS and Thomson Reuters with non-missing data on board characteristics		1586
Less: firms in financial and utilities industries		442
Less: firms with missing observations for our dependent variable		370
Less: firms with missing data on our control variables		20
Final sample		754
Panel B: Sample distribution by industries		
Fama-French industries	Number of Firms	Percent of Firms (%)
Business services	81	10.74
Retail	53	7.03
Machinery	41	5.44
Petroleum and natural gas	41	5.44
Electronic equipment	40	5.31
Chemicals	36	4.77
Wholesale	33	4.38
Transportation	32	4.24
Food products	26	3.45
Pharmaceutical products	25	3.32
Construction materials	24	3.18
Restaurants, hotels, motels	24	3.18
Automobile and trucks	22	2.92
Computers	21	2.79
Consumer goods	19	2.52
Medical equipment	19	2.52
Steel works etc	19	2.52
Construction	18	2.39
Measuring and control equipment	17	2.25
Communication	17	2.25
Healthcare	16	2.12
Other industries	130	17.24
Total	754	100.00

ISS: institutional shareholder services.

*Note*. This table provides the sample selection procedure in panel A and the sample distribution by Fama-French industries in panel B.

Panel B of Table 1 provides the sample distribution across the Fama and French (1997) industries. The statistics suggest that each industry consists of less than 11% of the final sample. We observe that each of the top five industries consists of more than 5% of the sample firms. The top five industries are Business Services, Retail, Machinery, Petroleum and Natural gas and Electronic Equipment. Each of the next 16 industries represents more than 2% but less than 5% of the sample firms. The remaining Fama and French (1997) industries in total represent 17.24%, whereby each industry represents less than 2% of the sample firms. The sample distribution suggests that the sample firms are nearly evenly distributed across the industries.

Table 2 reports the descriptive statistics on all key variables. The average cumulative excess stock return suggests that our sample firms experienced a 58% decline in cumulative excess stock returns during the COVID-19 collapse period. The average board tenure suggests that our sample firms’ outside directors have been serving for an average of 8.1 years on the firms’ board as of the 2019 fiscal year. This ranges from 6 to 10 years in quartiles one and three, respectively. On average, the executive directors’ board tenure is 9.5 years, which ranges from 3 years in quartile one to 13 years in quartile three.

**Table 2.** Descriptive statistics.

Variables	Mean	Median	Std	Q1	Q3
$CumExcRet_{t+1}$	−0.5815	−0.5835	0.1353	−0.6709	−0.4988
IndDirTenure	8.0610	7.7208	3.4466	5.7500	9.9091
*ExecDirTenure*	9.4655	6.1667	9.0594	3.0000	13.0000
*BusyBoard*	0.0862	0.0000	0.2809	0.0000	0.0000
*BoardSize*	9.5199	9.0000	1.9236	8.0000	11.0000
*CEODuality*	0.3674	0.0000	0.4824	0.0000	1.0000
*PropFD*	0.2594	0.2500	0.1058	0.2000	0.3077
*BDindependence*	0.8179	0.8571	0.1049	0.7778	0.9000
*CEOinsider*	0.0743	0.0000	0.2624	0.0000	0.0000
*AvDirAge*	62.5696	62.3693	3.6503	60.2500	64.7273
*InsideOWN*	0.0463	0.0078	0.0977	0.0026	0.0308
*CEOanotherFirm*	0.3249	0.0000	0.4687	0.0000	1.0000
*PropFQDir*	0.2694	0.2500	0.1349	0.1538	0.3750
*MVE*	8.5003	8.3267	1.7439	7.2923	9.6100
*MTB*	3.3338	2.5750	9.3699	1.4983	4.7611
*LEV*	0.4563	0.2360	0.7582	0.0924	0.4690
*SA*	−4.2809	−4.4035	0.3782	−4.6369	−4.0359
*CASH*	0.1063	0.0575	0.1422	0.0268	0.1303
*AG*	0.1134	0.0566	0.2712	−0.0081	0.1453
*ROA*	0.0747	0.0726	0.0752	0.0395	0.1111
*AD*	0.0183	0.0000	0.1353	0.0000	0.0089
*NS*	−0.0021	−0.0007	0.0653	−0.0247	0.0069
*DIVYield*	0.0220	0.0137	0.0930	0.0046	0.0249
*IO*	0.8635	0.8855	0.1510	0.7874	0.9572
*MOMENTUM*	−0.9933	−0.9933	0.0020	−0.9944	−0.9922

*Note*. This table provides the descriptive statistics on the main variables. The definition of all variables is provided in Appendix 1.

The descriptive statistics show that around 9% of our sample boards have majority of outside directors serving on at least three other boards of public firms. Our sample firms have, on average, 10 directors and it ranges from 8 in quartile one to 11 directors in quartile three. On average, the CEO is also the Chairperson of the board in 37 percent of the sample firms. Our sample firms, on average, have 26% of female directors on the board. On average, 82% of the directors are independent outside directors. In 7% of the sample firms, the CEO is the only executive director on the board. The average age of the directors is 63 years, and it ranges from 60 in quartile one to 65 years in quartile three. On average, 4.6% of common outstanding stocks of our sample firms are owned by the directors, 32% of our sample firms have at least one outside director employed as a CEO of another firm, and 27% of directors on the board are financially qualified directors.

Furthermore, we observe the following descriptive statistics on the firm characteristics. The average log market value of equity of 8.5 at the end of 2019 calendar year equates to approximately $4.9 billion. The market-to-book value of equity suggests that our sample firms’ market value is, on average, 3.3 times more than their book value. On average, the long-term debt is 46% of the market value. The descriptive statistics show that the average size-age index is −4.3. We also observe that, on average, cash and cash equivalents are 11% of the market value. The sample firms experienced, on average, an 11% growth in their assets and were profitable with 7% return on assets in 2019. On average, the sample firms spend approximately 2% of its market value on advertising expenditures. Our sample firms, on average, had a small decrease in shares outstanding in 2019. The dividend yield is, on average, 2.2%. On average, 86% of common stocks of the sample firms are owned by institutional investors. The cumulative log excess stock returns of our sample firms in 2019 was −99.33%. This is moderately lower than that reported by Fahlenbrach et al. (2021).^5^

We provide the correlation matrix of the key variables in Table 3. We observe a positive correlation between outside directors’ average board tenure and cumulative excess stock returns. The correlation matrix also shows significant correlations between a few other board characteristics and cumulative excess stock returns, and between some proxies of firm characteristics and cumulative excess stock returns. We discuss the association between the control variables and cumulative excess stock returns in the next section upon considering them in a multi-variate regression. The correlations among many control variables are significant. However, the correlations are not large enough for multicollinearity to be an issue. We also examine the variance inflation factors (VIFs) and find that the maximum VIF is 2.4 for the market value of equity (untabulated). The VIFs are far below the threshold of 10, beyond which multicollinearity may be a concern.

**Table 3.** Correlation matrix.

Variables	*V1*	*V2*	*V3*	*V4*	*V5*	*V6*	*V7*	*V8*	*V9*	*V10*	*V11*	*V12*	*V13*
*V1: $CumExcRet_{t+1}$ *													
*V2: IndDirTenure *	**0.13**												
*V3: ExecDirTenure*	**0.14**	**0.35**											
*V4: BusyBoard*	−0.06	−**0.09**	−**0.08**										
*V5: BoardSize*	−0.01	−**0.08**	−**0.15**	−0.01									
*V6: CEODuality*	**0.09**	**0.09**	**0.27**	−0.04	0.01								
*V7: PropFD*	0.01	−**0.20**	−**0.12**	0.04	**0.18**	0.04							
*V8: BDindependence*	−**0.07**	−**0.27**	−**0.33**	0.05	**0.17**	**0.12**	**0.26**						
*V9: CEOinsider*	**0.10**	0.02	**0.19**	−0.02	−**0.10**	**0.13**	0.04	0.07					
*V10: AvDirAge*	0.07	**0.52**	**0.42**	−**0.11**	−**0.10**	**0.15**	−**0.17**	−**0.14**	0.01				
*V11: InsideOWN*	0.06	**0.26**	**0.41**	−**0.08**	−**0.17**	**0.08**	−**0.16**	−**0.44**	0.01	**0.23**			
*V12: CEOanotherFirm*	0.02	−**0.08**	−**0.18**	−0.03	**0.26**	0.07	**0.13**	**0.20**	0.02	−**0.20**	−**0.18**		
*V13: PropFQDir*	−0.06	0.03	−0.03	0.04	−**0.25**	0.06	−0.01	**0.13**	−0.01	**0.09**	−**0.08**	0.02	
*V14: MVE*	**0.21**	−**0.08**	−**0.08**	0.05	**0.47**	**0.12**	**0.20**	**0.14**	−0.03	−0.06	−**0.22**	**0.27**	−**0.12**
*V15: MTB*	**0.09**	0.05	0.04	−0.01	0.05	0.00	0.04	−0.03	−0.02	0.04	0.03	0.00	−0.07
*V16: LEV*	−**0.25**	−**0.12**	−**0.10**	0.01	−0.06	−0.03	−0.03	0.03	−0.03	0.01	−0.02	−**0.09**	**0.11**
*V17: SA*	−0.03	−**0.17**	−0.02	0.03	−**0.28**	−**0.16**	−**0.13**	−**0.14**	−0.06	−0.05	**0.08**	−**0.19**	0.06
*V18: CASH*	−**0.12**	−0.05	−0.01	0.04	−**0.11**	−0.02	−0.01	0.04	0.03	**0.07**	0.06	−**0.09**	0.03
*V19: AG*	−0.04	−0.03	0.06	−0.02	−0.01	0.02	0.04	−0.06	0.02	−0.02	0.01	−0.04	−0.01
*V20: ROA*	**0.26**	**0.11**	**0.09**	−0.05	**0.11**	0.05	0.06	−0.04	−0.02	0.00	0.03	0.04	−0.05
*V21: AD*	−**0.09**	0.05	−0.03	−0.01	−0.05	−0.02	0.05	0.00	−0.01	0.02	0.00	−0.03	**0.09**
*V22: NS*	−0.05	−**0.09**	0.01	−0.01	−0.05	0.01	−0.01	−**0.07**	−0.03	0.05	0.02	−0.07	−0.03
*V23: DIVYield*	−0.06	−**0.08**	−0.02	−0.01	0.00	0.07	0.04	0.03	0.00	−0.01	0.00	−0.02	0.02
*V24: IO*	−**0.23**	−**0.15**	−**0.20**	0.01	−**0.12**	−**0.13**	0.03	**0.17**	0.01	−**0.18**	−**0.29**	−0.04	0.04
*V25: MOMENTUM*	0.06	−0.01	0.01	−0.00	0.08	0.00	−0.01	−0.01	0.02	−0.03	−0.02	0.04	−0.05
Variables	*V14*	*V15*	*V16*	*V17*	*V18*	*V19*	*V20*	*V21*	*V22*	*V23*	*V24*	*V25*	
*V14: MVE*													
*V15: MTB*	**0.10**												
*V16: LEV*	−**0.35**	−**0.12**											
*V17: SA*	−**0.32**	−0.04	0.03										
*V18: CASH*	−**0.38**	−**0.09**	**0.44**	**0.08**									
*V19: AG*	0.07	0.01	−0.01	0.06	−**0.14**								
*V20: ROA*	**0.42**	**0.11**	−**0.32**	−**0.09**	−**0.34**	**0.15**							
*V21: AD*	−**0.19**	−0.01	**0.24**	0.05	**0.23**	−0.03	−**0.18**						
*V22: NS*	−**0.07**	−0.01	**0.15**	0.07	0.02	**0.58**	−**0.11**	−0.01					
*V23: DIVYield*	−**0.15**	−0.02	**0.28**	0.07	**0.25**	−0.03	−**0.09**	−0.01	0.01				
*V24: IO*	−**0.21**	−0.05	−0.02	**0.09**	−0.07	0.07	−0.03	−0.05	−0.04	−**0.11**			
*V25: MOMENTUM*	**0.30**	**0.08**	−**0.24**	−**0.11**	−**0.29**	0.03	**0.24**	−**0.18**	−0.05	−**0.17**	0.06		

*Note.* This table provides the Pearson correlation among the key variables. The definition of all variables is provided in Appendix 1.The correlation coefficient in bold are significant at least at the five percent level.

## 5. Main results

### 5.1. Effect of outside directors’ average board tenure

Our result from the multi-variate regression of model (1) is presented in column 1 of Table 4. We find an inverted U-shaped relation between outside directors’ board tenure and cumulative stock returns during the COVID-19 collapse period. The results show a positive (negative) coefficient on IndDirTenure ( $IndDirTenure^{2}$ ) that is significant at the 1 (5)% level. In terms of economic significance, our result suggests that, holding all other variables at mean, firms with outside directors having firm-specific accumulated knowledge over the past 8 years of their tenure experienced an average 58.18% decline in cumulative excess stock returns.^6^ However, holding all other variables at mean, firms with outside directors’ average board tenure of 4 years experienced an average 60.50% decline in cumulative excess stock returns. The difference in the predicted change in cumulative excess stock returns of 0.0232 (= −0.5818 − (−0.6050)) is economically significant. Since the standard deviation of cumulative excess stock returns is 0.1353, the change in cumulative excess stock returns from an average board tenure of four to eight years translates into a 17.15 (= 0.0232/0.1353) % increase compared with the standard deviation.

**Table 4.** The effect of outside directors’ average board tenure on cumulative excess stock returns during the COVID-19 collapse period.

Variables	With squared term [Column 1]		Without squared term [Column 2]		Subsample where IndDirTenure≤10 [Column 3]		Subsample where IndDirTenure>10 [Column 4]	
	Estimate	*t*-statistic	Estimate	*t*-statistic	Estimate	*t*-statistic	Estimate	*t*-statistic
Intercept	−4.4016	−1.69^*^	−4.3626	−1.66^*^	−3.4063	−1.18	−7.9969	−1.62
IndDirTenure	0.0082	2.58^***^	0.0031	2.05^**^	0.0087	3.00^***^	0.0009	0.38
$IndDirTenure^{2}$	−0.0002	−2.03^**^						
*ExecDirTenure*	0.0006	1.10	0.0006	1.06	0.0005	0.70	0.0002	0.27
*BusyBoard*	−0.0235	−1.57	−0.0246	−1.62	−0.0266	−1.47	0.0003	0.01
*BoardSize*	−0.0111	−4.37^***^	−0.0108	−4.24^***^	−0.0123	−3.97^***^	0.0000	0.00
*CEODuality*	0.0074	0.79	0.0083	0.86	0.0190	1.73^*^	−0.0106	−0.63
*PropFD*	−0.0140	−0.31	−0.0176	−0.39	−0.0284	−0.51	0.0841	1.05
*BDindependence*	0.0163	0.28	0.0273	0.47	−0.0207	−0.29	0.1270	1.51
*CEOinsider*	0.0018	0.10	0.0019	0.10	0.0063	0.29	0.0121	0.40
*AvDirAge*	−0.0008	−0.55	−0.0007	−0.50	−0.0034	−1.89^*^	0.0050	1.87^*^
*InsideOWN*	−0.0252	−0.48	−0.0239	−0.45	0.0016	0.02	−0.1333	−1.74^*^
*CEOanotherFirm*	0.0039	0.41	0.0032	0.35	0.0108	0.97	−0.0302	−1.68^*^
*PropFQDir*	−0.0309	−0.97	−0.0316	−0.99	–0.0395	−1.08	−0.0368	−0.57
*MVE*	0.0115	3.08^***^	0.0114	3.04^***^	0.0129	2.92^***^	0.0054	0.84
*MTB*	−0.0001	−0.21	−0.0001	−0.20	0.0001	0.34	−0.0002	−0.32
*LEV*	−0.0211	−2.26^**^	−0.0213	−2.30^**^	−0.0171	−1.66^*^	−0.0153	−0.53
*SA*	0.0132	1.05	0.0123	0.97	0.0112	0.82	0.0677	2.05^**^
*CASH*	−0.0027	−0.06	−0.0006	−0.01	0.0002	0.00	−0.1385	−0.97
*AG*	−0.0434	−2.23^**^	−0.0453	−2.34^**^	−0.0460	−2.09^**^	−0.0113	−0.35
*ROA*	0.2474	3.43^***^	0.2529	3.50^***^	0.1600	1.81^*^	0.3131	2.71^***^
*AD*	−0.0574	−2.66^***^	−0.0563	−2.61^***^	−0.3900	−2.80^***^	−0.0506	−2.15^**^
*NS*	0.0271	0.29	0.0270	0.29	0.0369	0.37	0.0688	0.38
*DIVYield*	−0.0382	−1.68^*^	−0.0425	−1.88^*^	−0.0465	−1.89^*^	0.4720	1.33
*IO*	−0.1251	−3.88^***^	−0.1189	−3.74^***^	−0.0993	−2.65^***^	−0.1850	−3.35^***^
*MOMENTUM*	−4.0306	−1.54	−3.9894	−1.51	−3.1861	−1.10	−7.3416	−1.48
Industry FE	Yes		Yes		Yes		Yes	
Adj. R-Square	0.3334		0.3315		0.2883		0.5085	
Sample size	754		754		579		175	

*Note.* This table provides the results from OLS regressions of cumulative excess stock returns during the COVID-19 collapse period on outside directors’ average board tenure, other board characteristics, firm characteristics, and industry fixed effects. The regression in Column 1 (2) includes (excludes) the squared term of outside directors’ average board tenure. The regression in Column 3 (4) is based on a subsample of firms where the average board tenure of outside directors is less than and equal to (more than) 10 years. The *t*-statistics are corrected for heteroskedasticity. The definition of all variables is provided in Appendix 1.

* , ^**^ and ^***^ denote significance at the 10%, 5% and 1% levels, respectively.

To identify the inflection point, we next estimate regression model (1) after excluding IndDirTenure and $IndDirTenure^{2}$ , and obtain the residuals. First, we examine the relation between cumulative excess stock returns’ residuals and outside directors’ tenure using locally weighted polynomial curve.^7^ Second, we obtain the derivative of cumulative excess stock returns’ residuals with respect to outside directors’ tenure. Third, we estimate outside directors’ tenure where the slope is zero. The result suggests that the inflection point is at around 10 years. This is consistent with the findings of Huang and Hilary (2018) who, using non-crisis period data, show that the inflection point ranges between 8 and 11 years for a relation between outside directors’ board tenure and Tobin’s Q residual.

**Figure 1.**
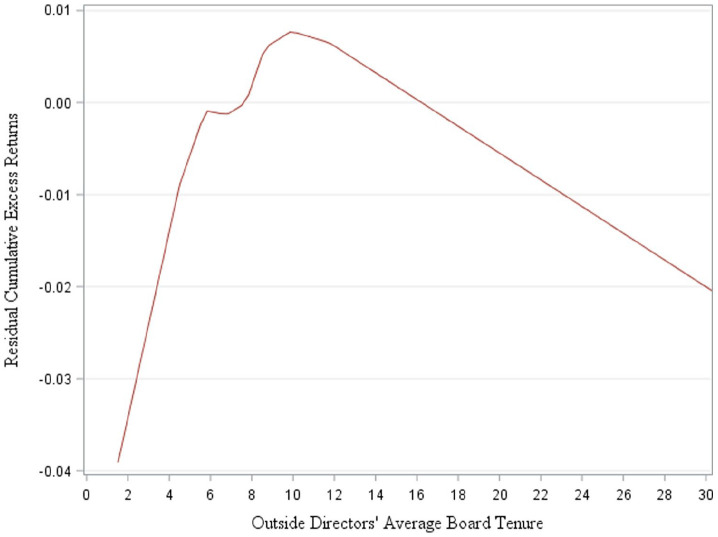
Residual cumulative excess returns and outside directors’ average board tenure. *Note*. This figure provides the graphical representation of the relation between outside director’s average board tenure and cumulative excess stock returns. We plot residual cumulative excess stock return on outside directors’ average board tenure using locally weighted polynomial curve. The residual is obtained from a regression of cumulative excess stock returns on the control variables, excluding the outside directors’ average board tenure and its squared term.

Next, we show the inverted U-shaped relation through a descriptive graphical presentation in Figure 1. The cumulative excess stock returns increase rapidly until around 6 years. The graph shows a dip in the cumulative excess stock returns from 6 to 7 years. The increase further continues to maximum cumulative excess stock returns at outside directors’ tenure equal to 10.1241 years. Subsequently, we observe a decline in the cumulative excess stock returns, but the slope for the decrease where tenure is more than 10 years is lower than the slope for the increase where tenure is less than 6 years.

Furthermore, we examine whether outside directors’ accumulated knowledge or the entrenchment effect dominates in our setting of the COVID-19 collapse period. Specifically, we re-estimate model (1) without $IndDirTenure^{2}$ . The result from column 2 shows a significant positive linear relation between outside directors’ board tenure and cumulative excess stock returns. This suggests that outside directors accumulated knowledge dominated the entrenchment effect. The result also suggests that outside directors’ accumulated knowledge benefitted firms during the COVID-19 collapse period.

In addition, we re-examine the linear relation using two subsamples. First, we use a subsample of firms with outside directors’ board tenure less than or equal to 10 years and provide our results in column 3. We find a significant positive relation between outside directors’ average board tenure and cumulative excess stock returns. Second, we use a subsample of firms with outside directors’ average board tenure of more than 10 years and provide our results in column 4. The result shows an insignificant relation between outside directors’ average board tenure and cumulative excess stock returns for firms with board tenure more than 10 years.

Our results from Table 4 collectively show a curvilinear (inverted U) relation between outside directors’ average board tenure and cumulative excess stock returns during the COVID-19 collapse period. The results suggest that outside directors’ accumulated knowledge in the form of firm-specific human and internal social capital benefitted the firm in terms of a lower decline in stock performance during the COVID-19 collapse period up to a threshold of 10 years of outside directors’ average board tenure. Our results also suggest that firms with outside directors’ average board tenure beyond 10 years experienced a stock performance that is, on average, slightly lower than other firms with outside directors’ average board tenure of about 10 years.

In terms of other board characteristics, the results show that executive directors’ average board tenure is not significantly associated with cumulative excess stock returns. We find a significant negative coefficient on *BoardSize*. The result shows that larger boards are associated with lower cumulative excess stock returns, suggesting that firms with larger boards suffered more during the COVID-19 collapse period. We do not find a significant coefficient on other board characteristics (i.e. Busy board, CEO duality, board gender diversity, board independence, CEO insider, directors’ average age, directors’ stock ownership, outside director being the CEO of another firm and proportion of financially qualified directors).

We observe significant coefficients on some firm characteristics that we control for in our empirical model. We find that firms with higher market value and return on assets are associated with higher cumulative excess stock returns. These results suggest that larger firms with higher profitability suffered less during the COVID-19 collapse period. Further, we find significant negative coefficients on leverage, asset growth, advertising expenditures, dividend yield and institutional shareholding. The results suggest that firms which had higher long-term debt and had relied on significant advertising in 2019 suffered more during the COVID-19 collapse period. These results are consistent with Fahlenbrach et al. (2021). The result also suggests that firms which had higher dividend yield and asset growth in 2019 suffered more during the COVID-19 collapse period. Firms with high institutional shareholdings also suffered more during the COVID-19 crisis. This result is consistent with Erkens et al. (2012), who show that firms with higher institutional ownership suffered more during the GFC because they took more risk prior to the crisis. We do not observe significant coefficients on other firm characteristics that we control for in our empirical model.

### 5.2. Effect of outside directors’ internal social capital

Our results for the tests of the second hypotheses (H2a and H2b) are provided in Table 5. Panel A provides the results for test of hypothesis H2a. We find that the curvilinear relation between outside directors’ average board tenure and cumulative excess stock returns is not significant for firms with a lower (first tercile) and moderate (second tercile) proportion of inside directors (columns 1 and 2). Our results show that the curvilinear relation is significant for firms with a higher (third tercile) proportion of inside directors (column 4). These results support H2a and suggest that outside directors’ internal social capital acquired through interactions with more inside directors was valuable during the health crisis. For example, in economic terms, considering the sample of firms with outside directors’ having a higher internal social capital (column 4), our results suggest a 28.7% increase in cumulative excess return compared with the standard deviation for boards with outside directors’ average board tenure from 4 to 8 years. Using locally weighted polynomial curve, our result in column 4 suggests that the inflection point is at 13.4 years. Hence, the result further suggests that outside directors’ extended board tenure (above 13.4 years) is detrimental to firms’ stock performance if outside directors have interactions with a higher proportion of inside directors.

**Table 5.** Effect of outside directors’ internal social capital on the relation between outside directors’ average board tenure and cumulative excess stock returns during the COVID-19 collapse period.

Variables	Low internal social capital subsample [Column 1]		Moderate internal social capital subsample [Column 2]		Moderate internal social capital subsample [Column 3]		High internal social capital subsample [Column 4]	
	Estimate	*t*-statistic	Estimate	*t*-statistic	Estimate	*t*-statistic	Estimate	*t*-statistic
***Panel A: Effect of the proportion of inside directors***								
Intercept	−2.9595	−0.81	−1.7806	−0.41			−7.8619	−1.38
IndDirTenure	0.0106	1.01	−0.0022	−0.19			0.0145	2.26^**^
$IndDirTenure^{2}$	−0.0006	−1.06	0.0005	0.69			−0.0004	−1.79^*^
Controls	Yes		Yes				Yes	
Industry FE	Yes		Yes				Yes	
Adj. R-Square	0.4096		0.2823				0.2925	
Sample size	284		231				239	
***Panel B: Effect of inside directors’ average board tenure***								
Intercept	−0.9619	−0.26	−2.5093	−0.54			−9.7052	−1.61
IndDirTenure≤10	0.0095	0.92	−0.0131	−1.02			0.0115	1.91^*^
$IndDirTenure^{2}$	−0.0002	−0.39	0.0006	0.87			−0.0003	−1.53
Controls	Yes		Yes				Yes	
Industry FE	Yes		Yes				Yes	
Adj. R-Square	0.2768		0.4495				0.2406	
Sample size	265		237				252	
***Panel C: Effect of the proportion of inside directors and inside directors’ average board tenure***								
Intercept	−3.0619	−0.84	−2.7947	−0.50	−11.7007	−1.53	−9.4859	−1.75^*^
IndDirTenure	0.0100	1.12	0.0150	1.07	0.0045	0.31	0.0105	1.83^*^
$IndDirTenure^{2}$	−0.0003	−0.57	−0.0008	−1.03	−0.0004	−0.50	−0.0003	−1.27
Controls	Yes		Yes		Yes		Yes	
Industry FE	Yes		Yes		Yes		Yes	
Adj. R-Square	0.3658		0.3226		0.1302		0.3590	
Sample size	254		162		123		215	

FE: fixed effects.

*Note.* This table provides the results from OLS regressions of cumulative excess stock returns during the COVID-19 collapse period on outside directors’ average board tenure, other board characteristics, firm characteristics, and industry fixed effects within subsamples based on proxies of the level of outside directors’ internal social capital. We use two proxies of outside directors’ internal social capital: (1) proportion of inside directors; and (2) inside directors’ average board tenure. Panel A provides the results for regressions within subsamples based on first, second and third terciles of the proportion of inside directors on the board in columns 1, 2 and 4, respectively. Panel B provides the results for regressions within subsamples based on the first, second and third terciles of inside directors’ average board tenure in columns 1, 2, and 4, respectively. Panel C provides the results for regressions within subsamples based on the proportion of inside directors and inside directors’ average board tenure. The subsample in column 1 (4) is based on firm-years with the proportion of inside directors and inside directors’ average board tenure below (above) their respective median. The subsample in column 2 (3) is based on firm-years with the proportion of inside directors below (above) its median and inside directors’ average board tenure above (below) its median. The *t*-statistics are corrected for heteroskedasticity. The definition of all variables is provided in Appendix 1.

* , ^**^ and ^***^ denote significance at the 10%, 5% and 1% levels, respectively.

We find similar results based on inside directors’ average board tenure as reported in Panel B. The results do not show a significant curvilinear relation between outside directors’ average board tenure and cumulative excess stock returns for boards with inside directors having a low (first tercile) or moderate (second tercile) average tenure (columns 1 and 2). However, our results show a significant relation between outside directors’ average board tenure and cumulative excess stock returns for boards with inside directors having a high (third tercile) average tenure (column 4). These results support H2b and suggest that outside directors’ internal social capital acquired through interactions with inside directors over more years was valuable during the health crisis. For example, in economic terms, the results in column 4 suggest a 23.4% increase in cumulative excess return compared with the standard deviation for boards with outside directors’ average board tenure from 4 to 8 years. In addition, our result in column 4 suggests that the inflection point is at 13.5 years.

Moreover, as an additional test, we examine the effect of outside directors’ internal social capital by forming subsamples of firms based on both the proportion of inside directors and their average board tenure. To avoid the loss of many firms within each subsample, we use the median of the proportion of inside directors and their average board tenure to divide the sample into two subsamples. As a result, we have four subsamples. Our first (last) subsample comprises firms with the proportion of inside directors below (above) the median and inside directors’ average board tenure below (above) the median. The regression results for these subsamples are provided in columns 1 and 4 of Panel C. The second and third subsamples comprise firms with the proportion of inside directors below (above) the median and inside directors’ average board tenure above (below) the median. The regression results for these subsamples are provided in columns 2 and 3. The results obtained based on this alternative sampling procedure to identify firms with potentially a higher versus a lower level of outside directors’ internal social capital support H2a and H2b. The result in column 4 also suggests an inflection point of 13.5 years.

## 6. Robustness checks and additional analyses

In this section, we discuss the results from several robustness checks and additional analyses. First, we use an alternative methodology and measure of firm performance. Second, we replicate our main analyses upon excluding firms from industries that are mostly represented in the sample and affected by the COVID-19 pandemic. Third, we replicate our main analyses using a propensity score-matched sample. Finally, we discuss results based on alternative time periods during the COVID-19 pandemic. Overall, the results from these robustness checks and additional analyses support our main findings.

### 6.1. Event study and alternative measure of firm performance

As our first robustness check, we use an event study methodology to examine investor reactions before and during the COVID-19 collapse period. We also examine investor reactions for the subsample of firms that have outside directors’ average board tenure below, within or above the optimal range of board tenure.

Since we focus on firm performance during the collapse period of COVID-19 that begins on 3 February 2020, we identify this date as our event date. Given that the effect of COVID-19 spans over a long period and several days before and after 3 February 2020, we use several event windows surrounding the event date to estimate cumulative average abnormal returns (*CAAR*). This approach is also consistent with our main analyses where we focus on cumulative excess stock returns. The measurement of *CAAR* is different from cumulative excess stock returns. Thus, *CAAR* represents an alternative measure of firm performance.

Consistent with prior studies (e.g. Brown and Warner, 1985; Naidu and Ranjeeni, 2021), we use the market model to estimate abnormal returns for each event window (Fama et al., 1969). First, we estimate the expected return ( $ER_{i,t}$ ) for each firm *i* at time *t* during the event window using the following equation

(2) $$ER_{i,t}={\hat{\alpha }}_{i}+{\hat{\beta }}_{i}R_{m,t}$$

where IndDirTenure represents the market return and is estimated as Standard & Poor’s (S&P) 500 composite index return at time *t*.^8^ The alpha ( ${\hat{\alpha }}_{i}$ ) and beta ( ${\hat{\beta }}_{i}$ ) parameters for each firm *i* are estimated using equation (2) and data from an estimation period of 250 trading days. The estimation period starts on 2 January 2019 and ends on 31 December 2019. The last day of the estimation period falls immediately before our event window, that is 22 trading days before the event date. We require a minimum estimation length of 127 trading days.

Second, we compute the abnormal returns ( $AR_{i,t}$ ) during the event window using equation (3). $R_{i,t}$ is the actual realized stock return of each firm *i* at time *t* within the event window.

(3) $$AR_{i,t}=R_{i,t}+ER_{i,t}$$

Third, we compute *CAAR* for each firm *i* and several event windows. To compute *CAAR*, we first measure the average abnormal returns for the full sample, and subsamples of firms with outside directors’ average board tenure below, within and above the optimal range of average board tenure. In our main analysis for the full sample (i.e. Table 4), we obtain an inflection point of 10 years and Huang and Hilary (2018) suggest that the optimal range of outside directors’ average board tenure is between 8 and 11 years. Thus, we use years between 8 and 11 as the optimal range of tenure. The average abnormal return is computed as the cross-sectional average of the abnormal returns on each trading day *t* within the event window for each sample. *CAAR* is computed by accumulating the average abnormal returns for an event window for each sample. To test the statistical significance of each *CAAR*, we use the standardized cross-sectional test approach (Boehmer et al., 1991).

We use the following event windows to compute *CAAR*: [−21, −10], [−9, −2], [−9, −1], [−1, 0], [0, + 1], [−1, + 1], [−9, + 13], [0, + 13], [0, + 34] and [+ 14, + 34]. Prior studies have used two approaches to examine the effect of COVID-19 in the US stock markets. Our approach for the main analyses is consistent with Fahlenbrach et al. (2021) whereby we focus on the collapse period of COVID-19. Ramelli and Wagner (2020) use data for the first quarter of 2020 and divide the sample into three periods labelled as Incubation (January 2 to 17), Outbreak (January 20 to February 21) and Fever (February 24 to March 20). The use of an event study approach allows us to use a combination of the two sampling approaches used in prior studies.

Each event window is defined as follows. [−21, −10] is the Incubation period ranging from Thursday January 2 to Friday January 17, 2020. [−9, −2] is part of the Outbreak period ending two days before the start the collapse period ranging from Tuesday 21 January to Thursday 30 January 2020. [−9, −1] is also part of the Outbreak period but ends a day before the collapse period on Friday 31 January 2020. [−1, 0] represents a day before and the event day, that is the first day of the collapse period (Friday 31 January and Monday 3 February). [0, +1] represents the event day and a day after the event day (Monday February 3 and Tuesday February 4). [−1, +1] represents a day before to a day after the start of the collapse period (Friday 31 January to Tuesday 4 February). [−9, +13] is the Outbreak period ranging from Tuesday 21 January to Friday 21 February 2020. [0, +13] is part of the Outbreak period falling within the collapse period ranging from Monday 3 February to Friday 21 February. [0, +34] is the collapse period ranging from Monday 3 February to Monday 23 March, which also corresponds to the trading days used to compute cumulative excess returns for our main analyses. [+14, +34] is the Fever period that also falls within the collapse period ranging from Monday 24 February to Monday 23 March 2020.

Table 6 provides the results on *CAAR* for each event window. The results show significant negative *CAAR* for all event windows during the first quarter of 2020. This is consistent with studies that suggest that COVID-19 had a significant negative effect on stock market performance (e.g. Ramelli and Wagner, 2020). The *CAAR* for firms with outside directors’ average board tenure below the optimal range of tenure (columns 2) is lower than that of firms within the optimal range (column 3) for most of the event windows. This is more apparent for longer event windows during the Outbreak, Fever and collapse periods. For example, the difference in *CAAR* for [−9, −2] in columns 2 and 3 for the Outbreak period falling before the collapse period is 1.29%. The difference in the Outbreak period that falls within the collapse period, [0, +13], is 1.69%. The difference in the Outbreak period, [−9, +13], is 2.76%. The difference in the Fever period, [+14, + 34], is 2.12%. The difference in the two groups in columns 2 and 3 for the collapse period [0, +34] is the largest, which is 3.78%.

**Table 6.** Effect of outside directors’ internal social capital on the relation between outside directors’ average board tenure and cumulative excess stock returns during the COVID-19 collapse period.

Event windows	Full sample [Column 1]		Subsample for firms below optimal range of outside directors’ tenure IndDirTenure<8 [Column 2]		Subsample for firms within optimal range of outside directors’ tenure 8≤IndDirTenure≤11 [Column 3]		Subsample for firms above optimal range of outside directors’ tenure IndDirTenure>11 [Column 4]	
	N	*CAAR*	N	*CAAR*	N	*CAAR*	N	*CAAR*
[−21, −10]	751	−1.47%	405	−1.73%	223	−1.70%	123	−0.16%
		−6.81^***^		−5.08^***^		−4.78^***^		−1.17
[−9, −2]	751	−2.13%	405	−2.69%	223	−1.40%	123	−1.63%
		−9.47^***^		−8.36^***^		−3.98^***^		−2.82^***^
[−9, −1]	751	−2.39%	405	−2.81%	223	−1.73%	123	−2.17%
		−10.54^***^		−8.48^***^		−4.86^***^		−4.04^***^
[−1, 0]	751	−0.42%	405	−0.28%	223	−0.52%	123	−0.72%
		−3.95^***^		−1.76^*^		−2.92^***^		−3.13^***^
[0, + 1]	751	−0.57%	405	−0.58%	223	−0.47%	123	−0.72%
		−3.85^***^		−2.68^***^		−1.91^*^		−2.09^**^
[−1, + 1]	751	−0.83%	405	−0.71%	223	−0.81%	123	−1.25%
		−5.65^***^		−3.16^***^		−3.35^***^		−4.11^***^
[−9, + 13]	751	−3.25%	405	−4.07%	223	−1.31%	123	−4.05%
		−8.32^***^		−6.74^***^		−3.49^***^		−3.57^***^
[0, + 13]	751	−0.86%	405	−1.26%	223	0.43%	123	−1.89%
		−3.12^***^		−2.49^**^		−1.00		−1.84^*^
[0, + 34]	751	−9.65%	405	−11.21%	223	−7.43%	123	−8.53%
		−8.23^***^		−6.72^***^		−3.77^***^		−2.95^***^
[+ 14, + 34]	750	−8.80%	404	−9.98%	223	−7.86%	123	−6.64%
		−7.65^***^		−6.39^***^		−3.52^***^		−2.49^**^

CAAR: cumulative average abnormal returns.

*Note*. This table provides the cumulative average abnormal returns (*CAAR*) for the full sample (column 1) and subsamples based on outside directors’ average board tenure. Column 2 (3) [4] provides *CAAR* for the subsample of firms that have outside directors’ average board tenure below (within) [above] the optimal range. We identify the first day of the COVID-19 collapse period as our event date, which is 3 February 2020. We use an estimation period of 250 trading days ending 22 days before the event date and requiring a minimum estimation length of 127 days. *CAAR* is reported for various event windows from 21 days before and 934 days after the event date. The *t*-statistics for each *CAAR* are reported in the second row for each event window. N represents the sample size.

* , ^**^ and ^***^ denote significance at the 10%, 5% and 1% levels, respectively.

A comparison of the *CAAR* between the subsample of firms with outside directors’ average board tenure within the optimal range (column 3) and above the optimal range (column 4) shows that the *CAAR* is larger for firms within the optimal range for most event windows. The difference in *CAAR* between these two groups within the Outbreak period before the collapse period is less than 0.5%. However, the difference in *CAAR* for the whole Outbreak period is 2.74%, and for the Outbreak period that falls within the collapse period is 2.32%. The difference between the two groups during the collapse period is 1.10%.

Collectively, the results from applying the event study methodology and using an alternative measure for firm performance, that is *CAAR*, provide support to our main results. The results suggest that the decline in firm performance during the initial stage of COVID-19 (i.e. Outbreak, Fever and collapse periods) is the lowest for firms with outside directors’ average board tenure between 8 and 11 years.

### 6.2. Industry exclusion test

The COVID-19 pandemic did not affect all industries in a similar fashion (Ramelli and Wagner, 2020). In our main analyses, we control for the industry effect by including industry-fixed effects in our regressions. In this section, we check the sensitivity of our results by excluding firms in selected industries. The industry median and average cumulative excess stock returns are around −58% for our sample. To ensure a balance in our approach to excluding firms from the sample for this test, we exclude some industries that have average cumulative excess returns below the median and some above the median. This approach controls for biasness in results that may occur if all firms that are mostly affected are excluded from the sample. Except for healthcare industry, we exclude other industries that are mostly represented in the sample (i.e. top five industries reported in Panel B of Table 1). Nevertheless, we also perform further analyses where we exclude firms based on their respective industry average cumulative excess returns. We provide the regression results in Table 7.

**Table 7.** Sensitivity test based on industries most represented in the sample.

Variables	With squared term [Column 1]		Without squared term [Column 2]		Subsample where IndDirTenure≤10 [Column 3]		Subsample where IndDirTenure>10 [Column 4]	
	Estimate	*t*-statistic	Estimate	*t*-statistic	Estimate	*t*-statistic	Estimate	*t*-statistic
***Panel A: Sample excludes Healthcare industry***								
Intercept	−4.2787	−1.62	−4.2473	−1.60	−3.5308	−1.21	−7.7461	−1.52
IndDirTenure	0.0081	2.52^>**^	0.0029	1.89^>*^	0.0084	2.83^>***^	−0.0002	−0.09
$IndDirTenure^{2}$	−0.0002	−2.02^>**^						
Controls	Yes		Yes		Yes		Yes	
Industry FE	Yes		Yes		Yes		Yes	
Adj. R-Square	0.3344		0.3325		0.2898		0.5109	
Sample size	738		738		566		172	
***Panel B: Sample excludes Business Services industry***								
Intercept	−3.8779	−1.53	−3.9084	−1.54	−2.5544	−0.88	−9.7283	−1.95^>*^
IndDirTenure	0.0078	1.98^>**^	0.0022	1.35	0.0058	1.98^>**^	−0.0002	−0.06
$IndDirTenure^{2}$	−0.0003	−1.75^>*^						
Controls	Yes		Yes		Yes		Yes	
Industry FE	Yes		Yes		Yes		Yes	
Adj. R-Square	0.3521		0.3511		0.2953		0.5382	
Sample size	673		673		515		158	
***Panel C: Sample excludes Retail industry***								
Intercept	−5.9655	−2.20^>**^	−5.8895	−2.16^>**^	−5.7716	−1.98^>**^	−5.1343	−0.99
IndDirTenure	0.0084	2.51^>**^	0.0035	2.27^>**^	0.0099	3.31^>***^	0.0026	1.01
$IndDirTenure^{2}$	−0.0002	−1.82^>*^						
Controls	Yes		Yes		Yes		Yes	
Industry FE	Yes		Yes		Yes		Yes	
Adj. R-Square	0.3433		0.3415		0.2931		0.5145	
Sample size	701		701		538		163	
***Panel D: Sample excludes Machinery industry***								
Intercept	−4.8040	−1.78^>*^	−4.7498	−1.75^>*^	−3.8958	−1.32	−7.2962	−1.47
IndDirTenure	0.0077	2.44^>**^	0.0029	1.89^>*^	0.0079	2.71^>***^	0.0019	0.76
$IndDirTenure^{2}$	−0.0002	−1.92^>*^						
Controls	Yes		Yes		Yes		Yes	
Industry FE	Yes		Yes		Yes		Yes	
Adj. R-Square	0.3383		0.3368		0.2931		0.5090	
Sample size	713		713		553		160	
***Panel E: Sample excludes Petroleum and Natural Gas industry***								
Intercept	−3.4939	−1.35	−3.4797	−1.34	−2.2756	−0.80	−8.4354	−1.70^>*^
IndDirTenure	0.0089	2.65^>***^	0.0038	2.46^>**^	0.0090	3.07^>***^	0.0003	0.12
$IndDirTenure^{2}$	−0.0002	−1.82^>*^						
Controls	Yes		Yes		Yes		Yes	
Industry FE	Yes		Yes		Yes		Yes	
Adj. R-Square	0.3041		0.3023		0.2617		0.4621	
Sample size	713		713		543		170	
***Panel F: Sample excludes Electronic Equipment industry***								
Intercept	−4.6906	−1.73^>*^	−4.6784	−1.71^>*^	−3.9552	−1.34	−6.0024	−1.07
IndDirTenure	0.0088	2.77^>***^	0.0035	2.10^>**^	0.0085	2.86^>***^	−0.0009	−0.30
$IndDirTenure^{2}$	−0.0003	−2.24^>**^						
Controls	Yes		Yes		Yes		Yes	
Industry FE	Yes		Yes		Yes		Yes	
Adj. R-Square	0.3265		0.3243		0.2780		0.5316	
Sample size	714		714		556		158	
***Panel G: Sample excludes five most represented industries (Business Services, Retail, Machinery, Petroleum and Natural Gas and Electronic Equipment)***								
Intercept	−4.9762	−1.76^>*^	−5.0492	−1.78^>*^	−5.2691	−1.78^>*^	8.6637	1.30
IndDirTenure	0.0124	1.90^>*^	0.0037	1.77^>*^	0.0060	1.78^>*^	−0.0017	−0.40
$IndDirTenure^{2}$	−0.0005	−1.48						
Controls	Yes		Yes		Yes		Yes	
Industry FE	Yes		Yes		Yes		Yes	
Adj. R-Square	0.3327		0.3314		0.2560		0.6045	
Sample size	482		482		376		106	

FE: fixed effects.

*Note*. This table provides the results from OLS regressions of cumulative excess stock returns during the COVID-19 collapse period on outside directors’ average board tenure, other board characteristics, firm characteristics, and industry fixed effects. The analysis uses subsamples upon excluding firms in industries that are most represented in the main sample. The title of each panel indicates the industry that is excluded from each test. The regression in column 1 (2) includes (excludes) the squared term of outside directors’ average board tenure. The regression in column 3 (4) is based on a subsample of firms where the average board tenure of outside directors is less than and equal to (more than) 10 years. The *t*-statistics are corrected for heteroskedasticity. The definition of all variables is provided in Appendix 1.

* , ^**^ and ^***^ denote significance at the 10%, 5% and 1% levels, respectively.

First, we check the sensitivity of our results upon excluding firms in the healthcare industry. Healthcare industry is one of the industries that is mostly affected by COVID-19. The industry average cumulative excess return is −60.95%. The results in Panel A show that they are consistent with our main findings. Second, we exclude firms in the business services industry. This industry has the highest representation in our sample. Also, the industry’s average cumulative excess return of −54.83% is below the median. Our results from the replication of the main analysis for a sample without firms in the business services industry reported in Panel B is consistent with our main findings.

Third, we exclude firms in the retail (machinery) industry that represents the second (third) highest in our sample. The industry’s average cumulative excess return is −60.34 (−60.49)%. This is below the sample median. The results in Panel C (D) are consistent with the main findings. Next, we exclude firms in the petroleum and natural gas industries. It represents the fourth largest in our sample and the second lowest in terms of average cumulative excess returns, which is −73.49%.^9^ We continue to find consistent results in Panel E. Also, we exclude firms in electronic equipment industry that is represented as the fifth largest in our sample. It has an average cumulative excess return of −52.29 percent, which is above the median. We find consistent results in Panel F. In Panel G, we show that the results hold if we exclude firms in the industries represented as top five in our sample. A drawback of this test is that it significantly reduces our sample size. Nevertheless, our results are consistent with the main findings.

Moreover, we exclude firms in terms of a set of industries most affected during the COVID-19 collapse period. For this approach, we do not individually exclude each industry because some industries have a very low representation in our sample (e.g. fabricated products). To conserve space, we discuss the results untabulated. We exclude firms in industries whose average cumulative excess return is below 70%. These industries are fabricated products, petroleum and natural gas and recreation. The sample representation of these industries is 1 firm, 41 and 4 firms, respectively. The industry average cumulative excess return is −81.76, −73.49 and −70.27, respectively. The results from replicating the main test upon excluding firms in these industries from our sample are consistent with the main findings ( IndDirTenure : Estimate = 0.0089; *t*-statistic = 2.67; $IndDirTenure^{2}$ : Estimate = −0.0003; *t*-statistic = −1.85).

Furthermore, we exclude firms in industries whose average cumulative excess return is between 65% and 70%. These industries are aircraft, textiles, entertainment, construction, restaurants, hotels, motels, and metallic and industrial metal mining. The sample size is 11, 3, 10, 18, 24 and 6, respectively. Our results are consistent with the main findings ( IndDirTenure : Estimate = 0.0084; *t*-statistic = 2.54; $IndDirTenure^{2}$ : Estimate = −0.0003; *t*-statistic = −2.16). Furthermore, we conduct a similar test upon excluding firms in industries whose average cumulative excess return is between 60% and 65% and continue to find consistent results. Our results from this test collectively suggest that our findings are not affected by the effect of COVID-19 on industry performance.

### 6.3. Propensity score-matching approach

Although we measure the outside directors’ average board tenure and all independent variables in year *t* − 1, that is 2019 fiscal year, endogeneity problems such as self-selection bias or omitted variables may still exist. It is possible that outside directors self-select to high-performing firms, and they choose to stay on the board of such firms as long as possible. Firms that have performed well prior to COVID-19 may perform better than other firms during the collapse period of COVID-19. Indeed, this is demonstrated by the coefficient on *ROA* that is positive and significant (see Table 4). Hence, the control for *ROA* in our analyses removes some of the effect of a relation between outside directors’ accumulated knowledge and firm performance in prior years whether it is due to self-selection bias or not. We also control for board characteristics and firm characteristics. Nevertheless, the endogeneity problem is not fully eliminated. Also, our main model may suffer from an omitted variable problem.

In this section, we examine our main analysis by using the propensity score matching approach to alleviate the endogeneity concerns. First, we estimate a conditional logistic regression of a dichotomous variable based on outside directors’ average board tenure. We code the dichotomous variable one if outside directors’ average board tenure is above the median, and zero otherwise. We regress the dichotomous variable on all control variables from Model (1). The untabulated results show that the model is reasonably fit. The Wald Chi-square and likelihood ratio are 143.73 and 271.81, respectively. Both are significant at the 1% level. The area under ROC curve is 0.8236 and Pseudo R^2^ is 40.36%.

Next, we use the coefficient from the logistic regression to determine a propensity score for each firm. We match each firm with high outside directors’ average board tenure to a unique firm with low outside directors’ average board tenure. Our matching is without replacement and with the neighbouring propensity score based on a calliper width of 0.01.^10^ The matching approach results in a sample of 348 firms. This is 46% of the main sample. Second, we compare the mean values on all control variables between the propensity-matched sample of high and low outside directors’ average board tenure. The untabulated results suggest that the mean values on the control variables are not significantly different across the two matched samples. Third, we replicate our main regression using the matched sample and provide the results in Table 8. The results confirm our main findings of the curvilinear relation between outside directors’ average board tenure and cumulative excess returns during the collapse period of COVID-19.

**Table 8.** Analyses based on propensity score-matched sample.

Variables	With squared term [Column 1]		Without squared term [Column 2]		Subsample where IndDirTenure≤10 [Column 3]		Subsample where IndDirTenure>10 [Column 4]	
	Estimate	*t*-statistic	Estimate	*t*-statistic	Estimate	*t*-statistic	Estimate	*t*-statistic
Intercept	−5.1615	−1.33	−4.4939	−1.14	−3.5205	−0.79	−8.0685	−1.69
IndDirTenure	0.0158	2.32^**^	0.0048	2.35^**^	0.0090	2.47^**^	0.0057	1.26
$IndDirTenure^{2}$	−0.0006	−1.89^*^						
Controls	Yes		Yes		Yes		Yes	
Industry FE	Yes		Yes		Yes		Yes	
Adj. R-Square	0.3306		0.3277		0.2554		0.6365	
Sample size	348		348		275		73	

FE: fixed effects.

*Note*. This table provides the results based on a propensity score-matched sample. The matched sample is formed based on a first-stage conditional logistic regression of dichotomous outside directors’ average board tenure on all control variables. We use a calliper width of 0.01 and matching without replacement. Before conducting these regressions, we check the mean values on all control variables between high and low outside directors’ average board tenure and they are not significantly different. Using the matched sample, we replicate our main analyses and the results appear in columns 1 to 4. The *t*-statistics are corrected for heteroskedasticity. The definition of all variables is provided in Appendix 1.

* , ^**^ and ^***^ denote significance at the 10%, 5% and 1% levels, respectively.

### 6.4. Alternative time periods during the COVID-19

Earlier analyses consider firm performance during several phases of COVID-19 in the first quarter of 2020, including the Incubation, Outbreak, Fever and collapse periods. COVID-19 has been continuing for several years. In this section, we consider the effect of outside directors’ average board tenure on cumulative excess returns during post collapse period of COVID-19. More specifically, we examine cumulative excess returns for three periods: (1) immediately post collapse defined as the time period between 24 March 2020 and 30 April 2020; (2) long-period post collapse defined as the time period between 24 March 2020 and 2 September 2020; (3) collapse and recovery period combined defined as the time period between 2 February 2020 and 2 September 2020.

We start accumulating excess returns for the post collapse period from 24 March 2020 because the collapse period ends on 23 March 2020. Also, the stimulus package in the US had an effect on stock returns from 24 March when the approval was learned by the market (Fahlenbrach et al., 2021). Subsequently, significant events occurred as the number of COVID-19 cases and deaths grew globally.^11^ The Federal Reserve also took actions to stimulate the economy. For example, on 30 April 2020, the Federal Reserve expanded the scope and eligibility for Main Street Lending Programme and the access to its Paycheck Protection Programme Liquidity Facility. Also, on the same day, the government’s guidelines on social distancing expired and states pushed for reopening plans.^12^ Hence, we end our first post collapse time period on April 30. In addition, on 4 September 2020, the Federal Reserve’s Main Street Lending Programme became fully operational. We end our second post collapse time period before this event on 2 September, consistent with section 3.3 of Fahlenbrach et al. (2021).

The cumulative excess returns rebounds in the post collapse time period that starts due to the government intervention via the stimulus package (Fahlenbrach et al., 2021). Since firms with outside directors’ average board tenure around the optimal range (i.e. around 10 years) suffered the least during the COVID-19 collapse period, we expect that these firms experienced the least rebound of stock returns. That is, we expect that firms which suffered more during the collapse period benefitted more from the stimulus package in the recovery period. We provide the results from replicating our main model (1) for alternative time periods in Table 9.

**Table 9.** Analyses based on alternative time periods during the COVID-19 pandemic.

Variables	Recovery period immediately post collapse [Column 1]		Longer recovery period [Column 2]		Collapse and recovery period combined [Column 3]	
	Estimate	*t*-statistic	Estimate	*t*-statistic	Estimate	*t*-statistic
Intercept	2.6590	0.44	13.6077	1.03	−9.5770	−1.32
IndDirTenure	−0.0150	−1.77^*^	−0.0230	−1.52	0.0073	1.26
$IndDirTenure^{2}$	0.0006	1.97^**^	0.0014	2.47^**^	0.0000	−0.16
Controls	Yes		Yes		Yes	
Industry FE	Yes		Yes		Yes	
Adj. R-Square	0.2904		0.2255		0.1322	
Sample size	752		752		752	

FE: fixed effects.

*Note*. This table provides the results from analyses based on alternative time periods during the COVID-19 pandemic. Column 1 provides the regression results for the recovery period immediately post collapse period. The time period is between 24 March 2020 (i.e. just after the stimulus package) and 30 April 2020. Column 2 provides the regression results for a longer recovery period where the time period is between 24 March 2020 and 2 September, 2020. Column 3 provides the regression results for the collapse and longer recovery period combined. The time period is between 2 February 2020 and 2 September 2020. For each column, the cumulative excess return is measured for the respective time period. We estimate the OLS regressions of cumulative excess stock returns for the respective time period on outside directors’ average board tenure, other board characteristics, firm characteristics, and industry fixed effects. The *t*-statistics are corrected for heteroskedasticity. The definition of all variables is provided in Appendix 1.

* , ^**^ and ^***^ denote significance at the 10%, 5% and 1% levels, respectively.

The results in column 1 show a U-shaped relation, and we estimate the inflection point as 9 years. Our results suggest that firms with outside directors’ average board tenure around nine benefitted the least immediately after the collapse period from the stimulus package. This is because such firms suffered the least during the collapse period. In column 2, over a longer post collapse period, we find similar results but mildly significant (only the squared term is statistically significant). Finally, we consider a holistic view of the effect of outside directors’ average board tenure on cumulative excess returns during the collapse and recovery period. The results in column 3 show insignificant coefficients. This result suggests that while firms with outside directors’ average board tenure around 10 years suffered the least during the collapse period, such firms also benefitted the least from the Federal Reserve’s stimulus package during the recovery period. Overall, the results suggest that outside directors’ accumulated knowledge has benefitted firms during the collapse period of COVID-19.

## 7. Implication for the Asia-Pacific region

Our findings have academic and practical relevance to Australian and Asia-Pacific region. COVID-19 had a significant effect on Australian Securities Exchange (ASX) listed companies. For example, Naidu and Ranjeeni (2021) show that the pandemic significantly and adversely affected firms from several sectors and of various sizes. Similarly, Rahman et al. (2020) examine ASX firms and find a negative stock market reaction to the announcement of the COVID-19 pandemic. They further show that the stock market reacted positively to one of the stimulus package − ‘JobKeeper’. Therefore, the findings of this study are relevant to the profession, regulators and companies in the Asia-Pacific region, including Australia.

Using a sample of 754 US firms, our descriptive statistics show that outside directors’ average board tenure is 8 years. Similarly, Huang and Hilary (2018) show that the average (median) board tenure is 8.22 (7.71) years using a sample of US firms between 1998 and 2010. Vafeas (2003) uses 483 firms listed on the 1994 Forbes list and shows that the median board tenure is 9.57 years. The distribution of board tenure differs for firms listed on the ASX. For example, He et al. (2020) use a sample of 867 ASX firms or 6020 firm-years between 2001 and 2014, and show that the average (median) board tenure is 6.6 (5.8) years. Similarly, Sun et al. (2022) use a sample of top 171 ASX firms and show that the average board tenure is 6.34 years. The difference in the statistics on the average board tenure between US and Australian firms and given that the average board tenure for ASX firms is far below the optimal range raises concern for regulations on board tenure and firms in Australia.

Our results are relevant to regulators and stakeholders of firms in the Asia-Pacific region because it highlights the beneficial effects of outside directors’ accumulated knowledge of the firm during the pandemic. Our results also highlight that optimal firm performance is achieved at about 10 years of outside directors’ board tenure. The studies that use ASX firms as their sample show that the average or median board tenure for Australian firms is below the optimal level. It is possible that the optimal level of board tenure for Australian firms is different from that of US firms. However, this is currently not known and future research may examine this using a sample of ASX firms. Our results imply that Australian firms may benefit if stakeholders allow outside directors to serve on the firm for around 10 years.

## 8. Conclusion

Corporate boards play a key role in advising and monitoring managers even during a crisis. Outside directors bring a wealth of expertise and experience to add value to the board deliberations and strategic decision-making. However, for outside directors to utilize their expertise and experiences efficiently and effectively, they require firm-specific knowledge in the forms of firm-specific human capital and internal social capital. We examine this during the COVID-19 health crisis.

Our research suggests that during the COVID-19 collapse period, US firms benefitted from outside directors’ accumulated knowledge of the firm. More specifically, our results suggest that outside directors’ higher accumulated knowledge contributed to investors experiencing a lower decline in cumulative excess stock returns during the COVID-19 crisis for firms with outside directors’ average board tenure of less than or equal to 10 years. We also find that this relation is profound for outside directors with a higher internal social capital. Our results suggest that outside directors’ internal social capital accumulated through their interactions with more inside directors over more years is useful for them in utilizing their human capital to contribute to board effectiveness. Our results are robust to a battery of sensitivity tests.

Our study contributes to the research on firm strategies that benefitted firms during the COVID-19 health crisis, corporate governance during a crisis, and the effect of outside directors’ firm-specific accumulated knowledge. Our findings have implications for regulators who are considering a limit on board tenure due to the trade-off between accumulated knowledge and compromised monitoring. The findings also have implications for the debate on the trade-off between board independence and management friendliness. Furthermore, the findings are relevant for regulators and stakeholders of firms in the Asia-Pacific region.

## Appendix 1. Definition and measurement of variables

**Table:**

Variables	Definition and measurement
***Dependent variables***	
$CumExcRet_{t+1}$	Represents cumulative excess stock returns. We use the log daily excess returns to compute the cumulative excess stock returns. Log daily excess returns represent the natural logarithm of one plus the stock return less 1-month daily Treasury bill rate. We cumulate the log daily excess returns over the COVID-19 collapse period
***Test variables***	
IndDirTenure	Is the average number of years since the outside directors have been serving on the firm’s board of directors
***Control variables***	
*ExecDirTenure*	Is the average number of years since the executive directors have been serving on the firm’s board of directors
*BusyBoard*	Is a dichotomous variable that is coded one for firms which have the majority of outside directors holding at least three directorships in other public firms, and zero otherwise
*BoardSize*	Is the number of directors serving on the board
*CEODuality*	Is a dichotomous variable that is coded one if the CEO is also the Chairman of the board, and zero otherwise
*PropFD*	Represents board gender diversity and is computed as the proportion of female directors on the board
*BDindependence*	Is the proportion of independent directors on the board
*CEOinsider*	Is a dichotomous variable that is coded one if the CEO is the only insider on the board, and zero otherwise
*AvDirAge*	Is the average age of directors on the board
*InsideOWN*	Is the proportion of common stock owned by directors
*CEOanotherFirm*	Is a dichotomous variable that is coded one if at least one independent director is employed as a CEO of another firm, and zero otherwise
*PropFQDir*	Is the proportion of financially qualified directors on the board
*MVE*	Is the natural logarithm of the market value as at the last trading day of 2019
*MTB*	Is the market value of equity divided by the book value of equity
*LEV*	Is the long-term debt deflated by the market value of equity
*SA*	Proxies for financial constraints and represents the Size-Age index of Hambrick and Fukutomi (1991)
*CASH*	Is computed as cash and equivalents deflated by the market value of equity
*AG*	Represents asset growth and is computed as the change in total assets divided by lagged total assets
*ROA*	Represents return on assets and is computed as the income before extraordinary items plus interest expense, deflated by lagged total assets
*AD*	Is the advertising expenditure deflated by the market value of equity
*NS*	Is the change in the natural logarithm of split-adjusted shares outstanding
*DIVYield*	Represents dividend yield and is computed as total dividend per share, deflated by stock price
*IO*	Represents the proportion of common stocks owned by institutional investors
*MOMENTUM*	Is the cumulative excess stock returns over the 2019 calendar year

## Notes

1. Sutherland (2020) suggests that the collective wisdom and experience of directors is important to shape a firm’s response to a crisis. Cohn (2020) in a Forbes article reports that ‘board needs to be available as a group or individually to provide advice and counsel based on their expertise and past experience’.

2. We also compute the economic effect using alternative values for outside directors’ average board tenure. Our results suggest that an increase in outside directors’ average board tenure from two to four, four to six, six to eight and eight to 10 (10 is the inflection point) years resulted into a 10.3%, 9.2%, 8.0% and 6.8% increase in cumulative excess stock returns during the COVID-19 collapse period, respectively.

3. As such, we respond to a recent call by Zattoni and Pugliese (2021) by addressing the research question: ‘How can boards leverage the competencies and the experiences of all directors if not physically present in the board room?’ (p. 1408).

4. The first COVID-19 case was reported in December 2019 in Wuhan, the capital of Hubei Province in China (Shan and Tang, 2020). Due to the sudden increase in the COVID-19 cases to 571 in China by 23 January 2020, the Chinese Government declared the city of Wuhan to be in lockdown effective from 10 am on 24 January.

5. Fahlenbrach et al. (2021) reports the average momentum as −83.2%. The difference could be due to a smaller sample used in our study than that in Fahlenbrach et al. (2021). Our sample is smaller due to the availability of data on board characteristics from ISS.

6. We compute the percentage effect on cumulative excess stock returns using the coefficients on *IndDirTenure* and *IndDirTenure*^2^ reported in column 1 of Table 4 as follows. The average cumulative excess stock return for our sample is −58.15%t, and after removing the effect of outside directors’ average board tenure (8.0610), it is −63.46% [ $=-0.5815-(0.0082\times 8.0610+-0.0002\times 8.0610^{2})$ ]. We compute the −58.18% at outside directors’ average board tenure of 8 years as $-0.6346+(0.0082\times 8+-0.0002\times 8^{2}).$ Similarly, we compute −60.50% at outside directors’ average board tenure of 4 years as $-0.6346+(0.0082\times 4+-0.0002\times 4^{2})$ . Also, we observe that firms with outside directors’ average board tenure of 12 (16) years experienced an average 56.50 (55.46) % decline in cumulative excess stock returns during the COVID-19 collapse period.

7. We use the SAS procedure, proc loess.

8. Our sample firms are from the universe of S&P 500 companies as covered by ISS that we use to obtain data on board characteristics.

9. The industry with the lowest average cumulative excess return (−81.76%) is fabricated products. We do not test for the exclusion of this industry because it is represented by only one firm in our sample.

10. We also check our results using alternative calliper width of 0.05 and 0.10 and find consistent results.

11. The timeline of events related to COVID-19 is available at https://fraser.stlouisfed.org/timeline/covid-19-pandemic.

12. Timeline of events is available at https://www.washingtonpost.com/nation/interactive/2021/coronavirus-timeline/.

## References

[bibr1-03128962231166831] Adams RB Ferreira D (2007) A theory of friendly boards. The Journal of Finance 62: 217–250.

[bibr2-03128962231166831] Associated Press (2020) Senate Fails to Advance Covid-19 Rescue Package. Available at: https://triblive.com/news/politics-election/as-crisis-deepens-congress-close-on-economic-rescue-deal/ (accessed 21 July 2020).

[bibr3-03128962231166831] Aufreiter N (2021) The Board’s Role during Crisis and beyond. Available at: https://www.mckinsey.com/capabilities/strategy-and-corporate-finance/our-insights/the-boards-role-during-crisis-and-beyond (accessed 5 August 2022).

[bibr4-03128962231166831] Bisson P (2021) The Board’s Role during Crisis and beyond. Available at: https://www.mckinsey.com/capabilities/strategy-and-corporate-finance/our-insights/the-boards-role-during-crisis-and-beyond (accessed 5 August 2022).

[bibr5-03128962231166831] Boehmer E Masumeci J Poulsen AB (1991) Event-study methodology under conditions of event-induced variance. Journal of Financial Economics 30: 253–272.

[bibr6-03128962231166831] Brown JA Anderson A Salas JM , et al. (2017) Do investors care about director tenure? Insights from executive cognition and social capital theories. Organization Science 28: 471–494.

[bibr7-03128962231166831] Brown SJ Warner JB (1985) Using daily stock returns: The case of event studies. Journal of Financial Economics 14: 3–31.

[bibr8-03128962231166831] Burt RS (1992) Structural Holes. Cambridge, MA: Harvard University Press.

[bibr9-03128962231166831] Cohn A (2020) The role of the board of directors during crisis. Forbes. Available at: https://www.forbes.com/sites/alisacohn/2020/06/03/the-role-of-the-board-of-directors-during-crisis/?sh=1121b028583f

[bibr10-03128962231166831] Coleman JS (1988) Social capital in the creation of human capital. American Journal of Sociology 94: S95–S120.

[bibr11-03128962231166831] Ding W Levine R Lin C , et al. (2021) Corporate immunity to the COVID-19 pandemic. Journal of Financial Economics 141: 802–830. 34580557 10.1016/j.jfineco.2021.03.005 PMC8457922

[bibr12-03128962231166831] Erkens DH Hung M Matos P (2012) Corporate governance in the 2007–2008 financial crisis: Evidence from financial institutions worldwide. Journal of Corporate Finance 18: 389–411.

[bibr13-03128962231166831] Fahlenbrach R Rageth K Stulz RM (2021) How valuable is financial flexibility when revenue stops? Evidence from the COVID-19 crisis. The Review of Financial Studies 34: 5474–5521.

[bibr14-03128962231166831] Fama EF French KR (1997) Industry costs of equity. Journal of Financial Economics 43: 153–193.

[bibr15-03128962231166831] Fama EF Fisher L Jensen MC , et al. (1969) The adjustment of stock prices to new information. International Economic Review 10: 1–21.

[bibr16-03128962231166831] Federal Reserve (2020) Federal Reserve Announces Extensive New Measures to Support the Economy. Available at: https://www.federalreserve.gov/newsevents/pressreleases/monetary20200323b.htm (accessed 21 July 2020).

[bibr17-03128962231166831] Fischer HM Pollock TG (2004) Effects of social capital and power on surviving transformational change: The case of initial public offerings. Academy of Management Journal 47: 463–481.

[bibr18-03128962231166831] Francis BB Hasan I Wu Q (2012) Do corporate boards matter during the current financial crisis? Review of Financial Economics 21: 39–52.

[bibr19-03128962231166831] Grant RM (1996) Toward a knowledge-based theory of the firm. Strategic Management Journal 17: 109–122.

[bibr20-03128962231166831] Hambrick DC Fukutomi GD (1991) The seasons of a CEO’s tenure. Academy of Management Review 16: 719–742. 10115480

[bibr21-03128962231166831] He L He R Evans E (2020) Board influence on a firm’s long-term success: Australian evidence. Journal of Behavioral and Experimental Finance 27: 100327.

[bibr22-03128962231166831] Huang S Hilary G (2018) Zombie board: Board tenure and firm performance. Journal of Accounting Research 56: 1285–1329.

[bibr23-03128962231166831] Jebran K Chen S (2023) Can we learn lessons from the past? COVID-19 crisis and corporate governance responses. International Journal of Finance & Economics 28: 421–429.

[bibr24-03128962231166831] Judge W (2012) The importance of considering context when developing a global theory of corporate governance. Corporate Governance: An International Review 20: 123–124.

[bibr25-03128962231166831] Kor YY Sundaramurthy C (2009) Experience-based human capital and social capital of outside directors. Journal of Management 35: 981–1006.

[bibr26-03128962231166831] Koutoupis A Kyriakogkonas P Pazarskis M , et al. (2021) Corporate governance and COVID-19: A literature review. Corporate Governance 21: 969–982.

[bibr27-03128962231166831] Lin Z Zhao X Ismail KM , et al. (2006) Organizational design and restructuring in response to crises: Lessons from computational modeling and real-world cases. Organization Science 17: 598–618.

[bibr28-03128962231166831] McKinsey & Company (2021) The Board’s Role during Crisis and beyond. Available at: https://www.mckinsey.com/capabilities/strategy-and-corporate-finance/our-insights/the-boards-role-during-crisis-and-beyond (accessed 5 August 2022).

[bibr29-03128962231166831] Naidu D Ranjeeni K (2021) Effect of coronavirus fear on the performance of Australian stock returns: Evidence from an event study. Pacific-Basin Finance Journal 66: 101520.

[bibr30-03128962231166831] Narayan PK Phan DHB Liu G (2020) COVID-19 lockdowns, stimulus packages, travel bans, and stock returns. Finance Research Letters 38: 101732. 32843886 10.1016/j.frl.2020.101732 PMC7440077

[bibr31-03128962231166831] Rahman ML Amin A Al Mamun MA (2020) The COVID-19 outbreak and stock market reactions: Evidence from Australia. Finance Research Letters 38: 101832. 36569654 10.1016/j.frl.2020.101832 PMC9761195

[bibr32-03128962231166831] Ramelli S Wagner AF (2020) Feverish stock price reactions to COVID-19. The Review of Corporate Finance Studies 9: 622–655. 10.1093/rcfs/cfaa012 PMC7454848 40504179

[bibr33-03128962231166831] Reuters (2020) Factbox: What’s in the $2 Trillion US Senate Coronavirus Rescue Package. Available at: https://www.reuters.com/article/us-health-coronavirus-usa-bill-factbox/factbox-whats-in-the-nearly-2-trillion-u-s-senate-coronavirus-stimulus-idUSKBN21B37G (accessed 21 July 2020).

[bibr34-03128962231166831] Schmidt B (2015) Costs and benefits of friendly boards during mergers and acquisitions. Journal of Financial Economics 117: 424–447.

[bibr35-03128962231166831] Shan C Tang DY (2020) The value of employee satisfaction in disastrous times: Evidence from Covid-19. Available at: 10.2139/ssrn.3560919

[bibr36-03128962231166831] Sun YV Liu B Prodromou T (2022) The determinants of the COVID-19 related stock price overreaction and volatility. Studies in Economics and Finance 39: 125–149.

[bibr37-03128962231166831] Sutherland S (2020) COVID-19: 10 Ways Boards Can Help Guide the Business during the Crisis. Available at: https://www.ey.com/en_gl/covid-19/covid-19-10-ways-boards-can-help-guide-the-business-during-the-crisis

[bibr38-03128962231166831] Vafeas N (2003) Length of board tenure and outside director independence. Journal of Business Finance & Accounting 30: 1043–1064.

[bibr39-03128962231166831] Von Post R Pozen RC (2020) Boards can guide businesses through a crisis. MIT Sloan Management Review, 7 May. Available at: https://sloanreview.mit.edu/article/boards-can-guide-businesses-through-a-crisis/

[bibr40-03128962231166831] Wade J O’Reilly CA III Chandratat I (1990) Golden parachutes: CEOs and the exercise of social influence. Administrative Science Quarterly 35: 587–603.

[bibr41-03128962231166831] Walsh JP Seward JK (1990) On the efficiency of internal and external corporate control mechanisms. Academy of Management Review 15: 421–458.

[bibr42-03128962231166831] Zaremba A Kizys R Aharon DY , et al. (2020) Infected markets: Novel coronavirus, government interventions, and stock return volatility around the globe. Finance Research Letters 35: 101597. 32550842 10.1016/j.frl.2020.101597 PMC7240275

[bibr43-03128962231166831] Zattoni A Pugliese A (2021) Corporate governance research in the wake of a systemic crisis: Lessons and opportunities from the COVID-19 pandemic. Journal of Management Studies 58: 1405–1410.

[bibr44-03128962231166831] Zhu DH (2013) Group polarization on corporate boards: Theory and evidence on board decisions about acquisition premiums. Strategic Management Journal 34: 800–822.

